# Integrated functional analysis of endometrial transcriptome, microbiome, and immune cell profiling is needed to dissect mechanisms of uterine pathologies and implantation failure

**DOI:** 10.1111/aogs.14882

**Published:** 2024-06-12

**Authors:** Ganesh Acharya

**Affiliations:** ^1^ Division of Obstetrics and Gynecology, Department of Clinical Science, Intervention & Technology (CLINTEC), Karolinska Institutet and Center for Fetal Medicine Karolinska University Hospital Stockholm Sweden; ^2^ Women's Health and Perinatology Research Group, Department of Clinical Medicine UiT‐The Arctic University of Norway Tromsø Norway

Human endometrium is a unique dynamic tissue that is renewed regularly in reproductive age women by undergoing regular cyclical changes which involve shedding, regeneration, proliferation, and remodeling in response to hormones. Thus, it is not surprising that substantial changes occur in gene expression, composition of microbiota, and immune cell profile in the reproductive tract tissues including endometrium during physiological transitions, such as menstrual cycle, pregnancy, and aging, as well as in pathological conditions. Our ability to investigate transcriptome, microbiome, and immune cell profile as well as the potential molecular functions in which differentially expressed genes and microbiota are involved, and their interaction with immune system has improved substantially in recent years due to the development of high‐throughput technologies, bioinformatics, and artificial intelligence. Multiomics approach has been widely applied in biomedical research. However, scientific breakthroughs of clinical significance have been scarce in the field of gynecology and reproductive medicine.

An article by Bui et al.^1^ published in this issue of AOGS investigated the potential association of endometrial transcriptome profile of mid‐luteal phase endometrial biopsies with embryo implantation outcomes. Results were compared between implantation failure and success groups in the short‐term (after the second fresh IVF/ICSI cycle) in 33 women with ongoing pregnancy vs. 52 with no pregnancy and in long‐term (including all fresh and frozen cycles within 12 months) in 23 infertile women with a livebirth after ≤3 embryos transferred vs. 23 with no livebirth after ≥3 good quality embryos transferred (i.e., recurrent implantation failure group). The embryos were not screened for aneuploidy by preimplantation genetic testing in this study.

The authors found no clearly distinct endometrial transcriptome profile that was associated with either implantation failure or success in infertile women. Similar findings with no differences in gene expression profile in endometrial biopsies taken during the proliferative phase from women having livebirth (*n* = 9) vs. women with implantation failure (*n* = 9) following embryo transfer performed in the same cycle have been previously reported.^2^

Plethora of studies on endometrial transcriptomics, including a single‐cell transcriptomic atlas of the human endometrium during the menstrual cycle, have been published improving our understanding of endometrial gene expression changes in physiological and pathological processes.^3^ However, a recently published systematic review of transcriptomic studies of the human endometrium revealed inconsistently reported differentially expressed genes and concluded that “the large majority of reported differentially expressed genes do not advance the identification of underlying biological mechanisms.”^4^ Furthermore, endometrial receptivity is affected not only by its transcriptomic profile but also by interactions with microbiota and immune cells in the reproductive tract. Endometrial microbial composition can be changed by exogenous hormones, which are commonly used during IVF/ICSI treatment, and microbiota can modulate immune tolerance during periconceptional period and pregnancy.^5^, ^6^, ^7^ However, the complex interplay between host microbiota and immune system and confounding effect of other intrinsic and extrinsic factors is often ignored.

Endometriosis and adenomyosis are common gynecological disorders associated with considerable morbidity and characterized by ectopic implantation and growth of endometrium. The etiology is unknown, but both conditions appear to share similar pathophysiology,^8^ and immune dysregulation/dysfunction is common in both conditions.^8^, ^9^

Another study published in this issue of journal by Valdés‐Bango et al.^10^ evaluated the composition of microbiota in the vagina, endometrium, and gut of individuals with (*n* = 38) and without (*n* = 46) adenomyosis. The main findings were reduced diversity of gut microbiota in patients with adenomyosis, significant differences in composition of gut and vaginal microbiota compared with controls, and distinct microbiota profiles among individuals with internal and external adenomyosis. Although these findings suggest a potential association between microbiota and adenomyosis, molecular mechanisms are not elucidated.

The uterine microenvironment is maintained in dynamic balance by a coordinated interaction between hormones, endometrial transcriptome, microbiome, and immune system. Therefore, an integrated functional approach investigating all these aspects is required to address mechanisms of uterine pathologies and implantation failure.
